# Association between serum 25-hydroxyvitamin D and osteoarthritis prevalence in US adults: a cross-sectional study using NHANES 2007–2023

**DOI:** 10.1097/MS9.0000000000005552

**Published:** 2026-09-10

**Authors:** Ibrahim Khalil, Mohammed Hrebat, Nadjet Laouadi, Ahmed Osman Hassan Ali, Tareq Albaqali, Mahmoud Ali Khazbak, Meera Abu Eisheh, Mahmoud Raed Sarahen, Taher Abuawwad, Farrukh Ameer, Bilal Aqel, Mohammad Hujeirat, Manar Atrash, Noor Nader Rashed, Ahmed Abdelsamad

**Affiliations:** a Department of Emergency Medicine, Red Crescent Specialized Hospital, Hebron, Palestine; b Faculty of Medicine, Tanta University, Tanta, Egypt; c Cardiac Anesthesia Consultant, Heart Center, Aljouf Health Cluster, Al-Jouf, Saudi Arabia; d Critical Care Department, Dr. Soliman Fakeeh Hospital, Riyadh, Saudi Arabia; e Faculty of Medicine, Jordan University of Science and Technology, Irbid, Jordan; f Directorate General of Health Services, Oman Ministry of Health, Muscat, Oman; g Palestine Polytechnic University, Hebron, Palestine; h Faculty of Medicine, Mansoura University, Mansoura, Egypt; i School of Medicine, Fujian Medical University, Fuzhou, China; j Department of Adult Cardiology, Dr. Soliman Fakeeh Hospital, Fakeeh Care Group, Riyadh, Saudi Arabia; k Faculty of Medicine, Al-Quds University, Jerusalem, Palestine; l Department of Internal Medicine, Chaim Sheba Medical Center at Tel HaShomer, Ramat Gan, Israel; m An-Najah National University, Nablus, Palestine; n Department of Surgery II, University of Witten/Herdecke - Knappschaft Klinikum Recklinghausen, Recklinghausen, Germany

**Keywords:** 25-hydroxyvitamin D, cross-sectional study, dose-response, NHANES, osteoarthritis, restricted cubic splines, vitamin D

## Abstract

Osteoarthritis (OA) is a leading cause of chronic disability globally, and the role of vitamin D in OA pathogenesis remains controversial. This cross-sectional study analyzed data from 35 887 US adults aged ≥20 years across seven NHANES cycles (2007–2023), all using standardized LC-MS/MS for serum 25-hydroxyvitamin D [25(OH)D] measurement. Survey-weighted logistic regression, restricted cubic splines (RCS), subgroup analyses with interaction testing, E-value assessment, Baron–Kenny CRP mediation analysis, and temporal trend evaluation were performed. The survey-weighted OA prevalence was 26.2%. In the fully adjusted model, each 10 nmol/L increase in 25(OH)D was associated with 5.3% higher odds of OA (OR = 1.053; 95% CI: 1.037–1.069; *P* < 0.001). RCS analysis revealed a threshold effect above 50 nmol/L, plateauing at 100–125 nmol/L. Significant effect modification was observed by age (*P* < 0.001) and BMI (*P* = 0.004), with the strongest association among obese adults (OR = 1.058). CRP did not mediate the association (−4.7%). The E-value was 1.29. Results were robust across seven sensitivity analyses and consistent across all survey cycles (ORs: 1.032–1.088). Higher serum 25(OH)D was positively associated with OA prevalence in a non-linear fashion, likely reflecting reverse causation or health-seeking behavior (i.e., patients with OA proactively taking vitamin D supplements) rather than a causal harmful effect, consistent with null results from vitamin D supplementation trials for OA.

## Introduction

Osteoarthritis (OA) is the most prevalent type of arthritis and a major contributor to chronic disability worldwide^[^1,2^]^. The Global Burden of Disease (GBD) 2021 report estimated that 595 million individuals were affected by OA worldwide in 2020, accounting for 7.6% of the world’s population, with an age-standardized prevalence that increased by 48.6% since 1990^[^1^]^. The knee is the second most commonly affected joint worldwide, accounting for 61% of the global OA burden^[^1^]^. OA affects an estimated 32.5 million adults in the United States and is a leading contributor to healthcare utilization, work disability, and compromised quality of life^[^2^]^. OA is characterized by the progressive degradation of articular cartilage, remodeling of the subchondral bone, formation of osteophytes, and inflammatory changes in the synovium, arising from the complex interdependence between mechanical, metabolic, genetic, and inflammatory factors^[^2,3^]^. Advanced age, female sex, obesity, prior joint injury, and genetic predisposition are well-established risk factors for OA^[^2,3^]^.

HIGHLIGHTS

- In 35 887 U.S. adults from NHANES 2007–2023, higher serum 25(OH)D levels were positively associated with osteoarthritis prevalence (OR = 1.053 per 10 nmol/L increase).
- Restricted cubic spline analysis revealed a non-linear threshold effect above 50 nmol/L, plateauing at 100–125 nmol/L.
- The association was significantly modified by age (*P* < 0.001) and BMI (*P* = 0.004), and was strongest among obese adults.
- C-reactive protein did not mediate the association, and results were consistent across all seven NHANES cycles (2007–2023).
- The findings likely reflect reverse causation or health-seeking behavior and align with null results from vitamin D supplementation RCTs.

Vitamin D is a fat-soluble secosteroid hormone necessary for calcium and phosphorus homeostasis as well as skeletal integrity^[^4^]^. According to the Institute of Medicine, serum 25-hydroxyvitamin D [25(OH)D] concentrations <30 nmol/L, 30–49 nmol/L, and ≥ 50 nmol/L are defined as deficient, insufficient, and sufficient, respectively^[^4,5^]^. Vitamin D: Beyond the skeleton–pleiotropic actions of vitamin D in musculoskeletal tissues. Articular chondrocytes express the vitamin D receptor (VDR), through which the active vitamin D metabolite, 1,25-dihydroxyvitamin D, regulates proteoglycan synthesis, modulates matrix metalloproteinase (MMP) expression, and reduces pro-inflammatory cytokine signaling in purified joint tissues^[^6,7^]^.

Vitamin D has also been found to suppress the expression of MMP-1, MMP-3, MMP-9, and MMP-13 in chondrocytes^[^7^]^, which are all key mediators of cartilage damage in OA. Moreover, vitamin D modulates osteoblast and osteoclast activity, thereby affecting subchondral bone remodeling^[^6^]^.

Vitamin D deficiency is a major public health issue in the United States. According to the National Health and Nutrition Examination Survey (NHANES) data, 5–8% of the US adult population has serum 25(OH)D levels below 30 nmol/L (deficient), while an additional 18–24% have serum levels of 30–49 nmol/L (insufficient)^[^8,9^]^. There are marked disparities by race and ethnicity, with non-Hispanic Black adults showing significantly higher rates of deficiency than their non-Hispanic White counterparts^[^8,9^]^. Other factors that determine low vitamin D status include obesity, low socioeconomic status, and limited sun exposure^[^8^]^.

Although the association between vitamin D status and OA has been extensively explored, the findings are inconsistent, and the direction of this association remains unclear. Several observational studies and meta-analyses have suggested an association between serum 25(OH)D levels and the risk of radiographic knee OA progression^[^10,11^]^, cartilage volume loss^[^10^]^, and greater symptom severity. Meta-analytic evidence has also revealed that 56.7% of patients with knee OA are vitamin D deficient^[^12^]^. In contrast, randomized controlled trials of vitamin D supplementation have mostly failed to show beneficial effects on the structural progression of OA^[^13^]^.

In addition, based on a retrospective cross-sectional analysis of the National Health and Nutrition Examination Survey (NHANES) 2001–2018, Yu *et al* reported a paradoxical positive association between higher serum 25(OH)D concentrations and OA prevalence, particularly among obese individuals, with odds ratios of 1.25, 1.62, and 1.91 for each increase in 25(OH)D category (50–75, 75–100, and ≥100 nmol/L), respectively, compared to <50 nmol/L^[^14^]^. Nonetheless, that study focused on adults aged ≥40 years, included NHANES cycles that spanned changes in assay methods, and did not assess nonlinear dose–response relationships, inflammatory mediation pathways, or temporal consistency.

However, several important knowledge gaps remain in this field. First, the shape of the population-level dose–response curve between serum 25(OH)D and OA has not been adequately characterized using flexible nonlinear modeling approaches. Second, whether systemic inflammation assessed by hs-CRP mediates the 25(OH)D–OA relationship remains to be determined. Third, the extent to which this association is consistent across demographic subgroups and NHANES survey cycles has not been directly assessed in a single analysis and remains unclear. These gaps must be addressed to implement evidence-based clinical guidelines for vitamin D and musculoskeletal health.

Thus, the current study aimed to investigate the relationship between serum 25(OH)D concentration and self-reported OA prevalence in a nationally representative sample of U.S. adults using pooled data from seven consecutive NHANES cycles (2007–2023), all of which were conducted using the same LC-MS/MS assay to measure 25(OH)D. We specifically aimed to (1) characterize the association using continuous and categorical modeling of exposures; (2) characterize the non-linear dose–response relationship via restricted cubic splines (RCSs); (3) evaluate effect modification by age, sex, body mass index (BMI), and race/ethnicity; (4) assess whether C-reactive protein (CRP) mediates the observed association; and (5) examine the temporal stability of this association across survey cycles.

## Methods

### Study design and data source

This cross-sectional study used data from the National Health and Nutrition Examination Survey (NHANES), a program conducted by the National Center for Health Statistics (NCHS) of the Centers for Disease Control and Prevention (CDC). NHANES employs a complex, stratified, multistage probability sampling design to obtain nationally representative estimates of the civilian, non-institutionalized U.S. population^[^1^]^. We pooled data from seven consecutive cycles: 2007–2008, 2009–2010, 2011–2012, 2013–2014, 2015–2016, 2017–2018, and 2021–2023 (the 2019–2020 cycle was incomplete due to the COVID-19 pandemic). These cycles used a standardized LC-MS/MS method for serum 25(OH)D measurement, ensuring cross-cycle comparability. All NHANES protocols were approved by the National Center for Health Statistics (NCHS) Research Ethics Review Board, and all participants provided written informed consent^[^15^]^.

### Study population

Participants were included if they were aged ≥20 years and had complete data on serum 25(OH)D (LBXVIDMS), self-reported OA (MCQ160A), BMI (BMXBMI), sex (RIAGENDR), race/ethnicity (RIDRETH1), valid MEC sample weights (WTMEC2YR), primary sampling unit (SDMVPSU), and stratum (SDMVSTRA). The complete-case analysis yielded a final sample of 35 887 adult participants.

### Outcome assessment

The primary outcome was self-reported OA, ascertained from the NHANES medical conditions questionnaire (MCQ160A: “Has a doctor or other health professional ever told you that you had arthritis?”). This measure has been extensively used in NHANES-based epidemiological studies and has demonstrated acceptable validity in population surveillance.

### Exposure assessment

The primary exposure was serum 25(OH)D concentration (nmol/L; LBXVIDMS), measured using standardized LC-MS/MS calibrated to the National Institute of Standards and Technology (NIST) and Vitamin D Standardization Program (VDSP) standards. Serum 25(OH)D was modeled in three ways: (1) as a continuous variable per 10 nmol/L increment; (2) by quartiles (Q1–Q4, with Q4 as the reference); and (3) using RCSs for non-linear dose–response. Vitamin D status was categorized as deficient (<30 nmol/L), insufficient (30–49 nmol/L), sufficient (50–74 nmol/L), or optimal (≥75 nmol/L).

### Covariates

Covariates were selected a priori, including age (continuous, RIDAGEYR), sex (RIAGENDR), race/ethnicity (RIDRETH1), and BMI (continuous, BMXBMI). The fully adjusted model additionally included smoking (SMQ020), diabetes mellitus (DIQ010), and poverty–income ratio (INDFMPIR) as covariates. High-sensitivity C-reactive protein (hsCRP) was used for mediation analysis.

### Statistical analysis

All analyses incorporated the NHANES complex survey design features (SDMVSTRA, SDMVPSU, WTMEC2YR) using the ‘survey’ package in R version 4.3.3^[^16^]^. Four nested logistic regression models were constructed: Model 0 (crude), Model 1 (+ age, sex), Model 2 (+ race/ethnicity, BMI), and Model 3 (+ smoking, diabetes, PIR). Nonlinear dose–response relationships were examined using RCSs with four knots at the 5th, 35th, 65th, and 95th percentiles^[^17^]^. Subgroup analyses were stratified by age, sex, BMI, and race/ethnicity using multiplicative interaction. Seven sensitivity analyses were performed to test the robustness of our results. E-values were calculated to assess unmeasured confounding^[^18^]^. CRP mediation was assessed using the Baron and Kenny causal steps approach^[^19^]^. Temporal trends were evaluated using cycle-specific analyses. Statistical significance was set at *P* < 0.05. To account for the pooling of seven separate survey cycles, multi-cycle survey weights were calculated by dividing the original 2-year MEC weights (WTMEC2YR) by the total number of combined cycles (seven), in accordance with NCHS analytical guidelines, to ensure the estimates remain representative of the average US civilian non-institutionalized population over the 16-year study period.

## Results

### Study population and baseline characteristics

A total of 35 887 adults aged ≥20 years from seven consecutive NHANES cycles (2007–2008 through 2021–2023) met the inclusion criteria and were included in the final analytic sample. The survey-weighted prevalence of self-reported OA was 26.2% (*n* = 10 144 cases). The mean age of the study population was 49.7 years, and 52.2% were female. The racial/ethnic distribution reflected the nationally representative design: 42.3% non-Hispanic White, 19.2% non-Hispanic Black, 12.4% Mexican American, 10.6% Other Hispanic, and 12.5% Other/Multi-racial.

The mean serum 25(OH)D concentration was 67.5 nmol/L (SD: 28.3). Participants were classified into quartiles of serum 25(OH)D: Q1 (6–46 nmol/L; *n* = 8972), Q2 (46–64 nmol/L; *n* = 8972), Q3 (64–84 nmol/L; *n* = 8972), and Q4 (84–424 nmol/L; *n* = 8971). Participants in higher vitamin D quartiles were more likely to be Non-Hispanic White, older, have a lower BMI, and have lower CRP concentrations (Table 1). The prevalence of OA increased with higher vitamin D quartiles.

**Table 1:** Baseline demographic, clinical, and biochemical characteristics of study participants stratified by serum 25-hydroxyvitamin D [25(OH)D] quartiles, NHANES 2007–2023 (*N* = 35,887).

Quartile	N	Vitamin D Range (nmol/L)	Age Mean (SD)	Female (%)	BMI Mean (SD)	OA (%)	Vitamin D Mean (SD)
Q1	8972	6–46	45.2 (16.6)	52.7	30.8 (8.2)	21.8	33.8 (8.3)
Q2	8972	46–64	47.3 (16.9)	47	29.7 (6.8)	22.6	55.2 (5.1)
Q3	8972	64–84	50.9 (17.3)	48.8	28.8 (6.5)	28.2	73.0 (5.6)
Q4	8971	84–424	58.1 (17.0)	60.2	28.1 (6.3)	40.5	107.9 (25.6)

### Association between serum 25(OH)D and osteoarthritis

In the unadjusted model (Model 0), each 10 nmol/L increase in serum 25(OH)D was associated with a 11.9% increase in the odds of OA (OR = 1.119; 95% CI: 1.105–1.133; *P* < 0.001). After adjustment for age and sex (Model 1), the association was substantially attenuated but remained statistically significant (OR = 1.023; 95% CI: 1.010–1.036; *P* < 0.001). Further adjustment for race/ethnicity and BMI (Model 2) yielded an OR of 1.042 (95% CI: 1.027–1.057; *P* < 0.001). In the fully adjusted model (Model 3), which additionally controlled for smoking status, diabetes mellitus, and the poverty–income ratio, the association remained robust (OR = 1.053; 95% CI: 1.037–1.069; *P* < 0.001; *n* = 31 516) (Table 2).

**Table 2:** Survey-weighted multivariable logistic regression models evaluating the association between serum 25(OH)D (continuous per 10 nmol/L increase and by quartiles) and osteoarthritis prevalence among US adults, NHANES 2007–2023.

Model	OR (95% CI)	*P*-value	*N*
Model 0 (Crude)	1.119 (1.105–1.133)	<0.001	35 887
Model 1 (Age + Sex)	1.023 (1.010–1.036)	<0.001	35 887
Model 2 (+Race + BMI)	1.042 (1.027–1.057)	<0.001	35 887
Model 3 (Full)	1.053 (1.037–1.069)	<0.001	31 516

When serum 25(OH)D was modeled as quartiles with the highest quartile (Q4: 84–424 nmol/L) as the reference, a significant inverse dose–response pattern was observed. Compared with Q4, Q1 (6–46 nmol/L) had an OR of 0.696 (95% CI: 0.616–0.787; *P* < 0.001), Q2 (46–64 nmol/L) had an OR of 0.702 (95% CI: 0.625–0.790; *P* < 0.001), and Q3 (64–84 nmol/L) had an OR of 0.823 (95% CI: 0.736–0.921; *P* < 0.001). The *P*-value for the linear trend across quartiles was statistically significant (*P* < 0.001), indicating a monotonic dose–response relationship (Table 2).

### Non-linear dose–response relationship

RCS analysis with 4 knots placed at the 5th, 35th, 65th, and 95th percentiles of the vitamin D distribution (26.2, 53.6, 74.8, and 122.0 nmol/L, respectively) revealed a non-linear dose–response relationship between serum 25(OH)D and the odds of OA (Fig. 1). Using 50 nmol/L as the reference level, the predicted OR remained relatively stable at lower vitamin D concentrations (OR at 20 nmol/L: 1.04, 95% CI: 0.91–1.19; OR at 30 nmol/L: 1.01, 95% CI: 0.91–1.12; OR at 40 nmol/L: 0.99, 95% CI: 0.90–1.09). However, the odds of OA increased substantially at concentrations above 50 nmol/L, with the OR rising to 1.06 (95% CI: 0.97–1.16) at 60 nmol/L, 1.22 (95% CI: 1.12–1.33) at 75 nmol/L, and reaching a plateau at 1.36 (95% CI: 1.25–1.49) at 100 nmol/L and 1.37 (95% CI: 1.25–1.51) at 125 nmol/L (Fig. 1). This non-linear pattern suggests that concentrations above the commonly used sufficiency threshold of 50 nmol/L are associated with progressively higher odds of OA.

**Figure 1.**
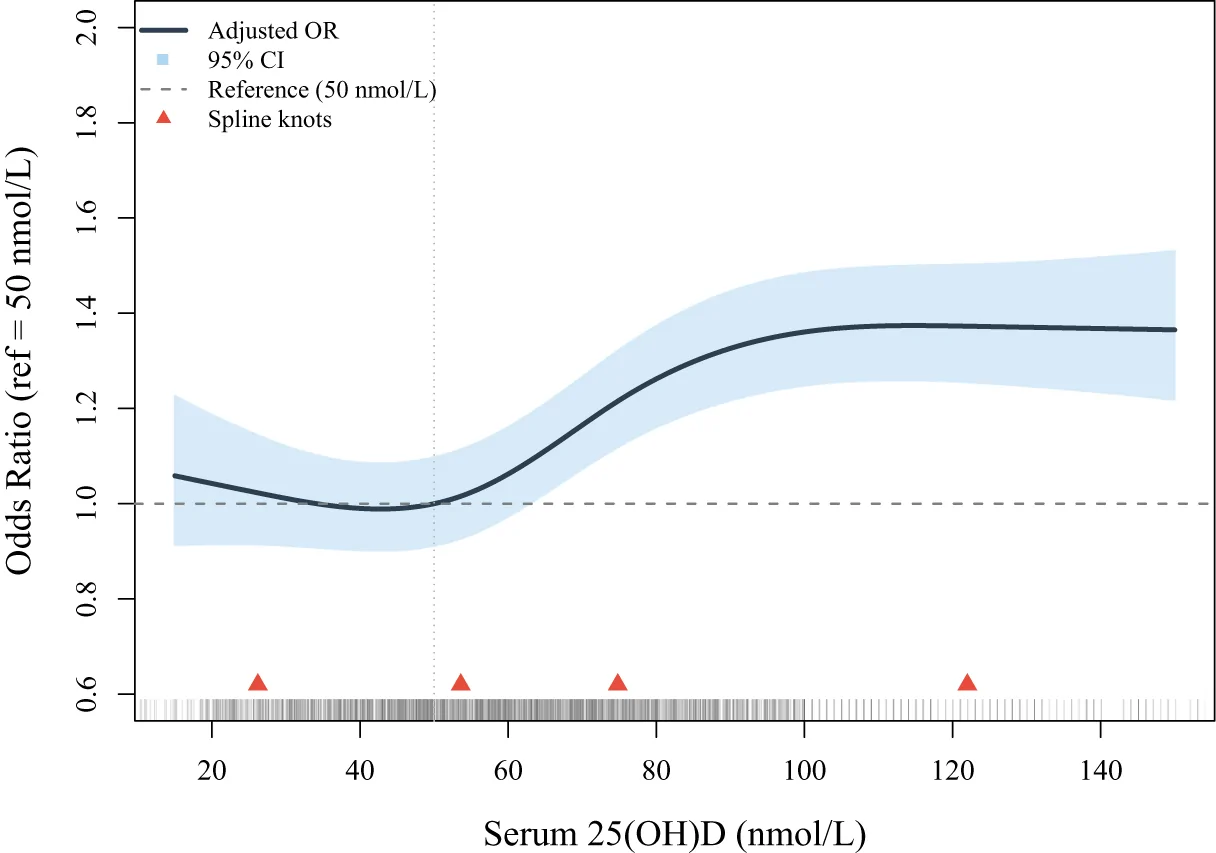
Non-linear dose-response (RCS with 4 knots) between serum 25(OH)D and odds of OA, adjusted for age, sex, race/ethnicity, and BMI, with reference at 50 nmol/L and the shaded area indicating the 95% CI, based on NHANES 2007–2023 (*N* = 35 887).

### Subgroup analyses and effect modification

Stratified analyses demonstrated that the positive association between serum 25(OH)D and OA was consistent across all demographic and clinical subgroups (Fig. 2; Table 3).

**Figure 2.**
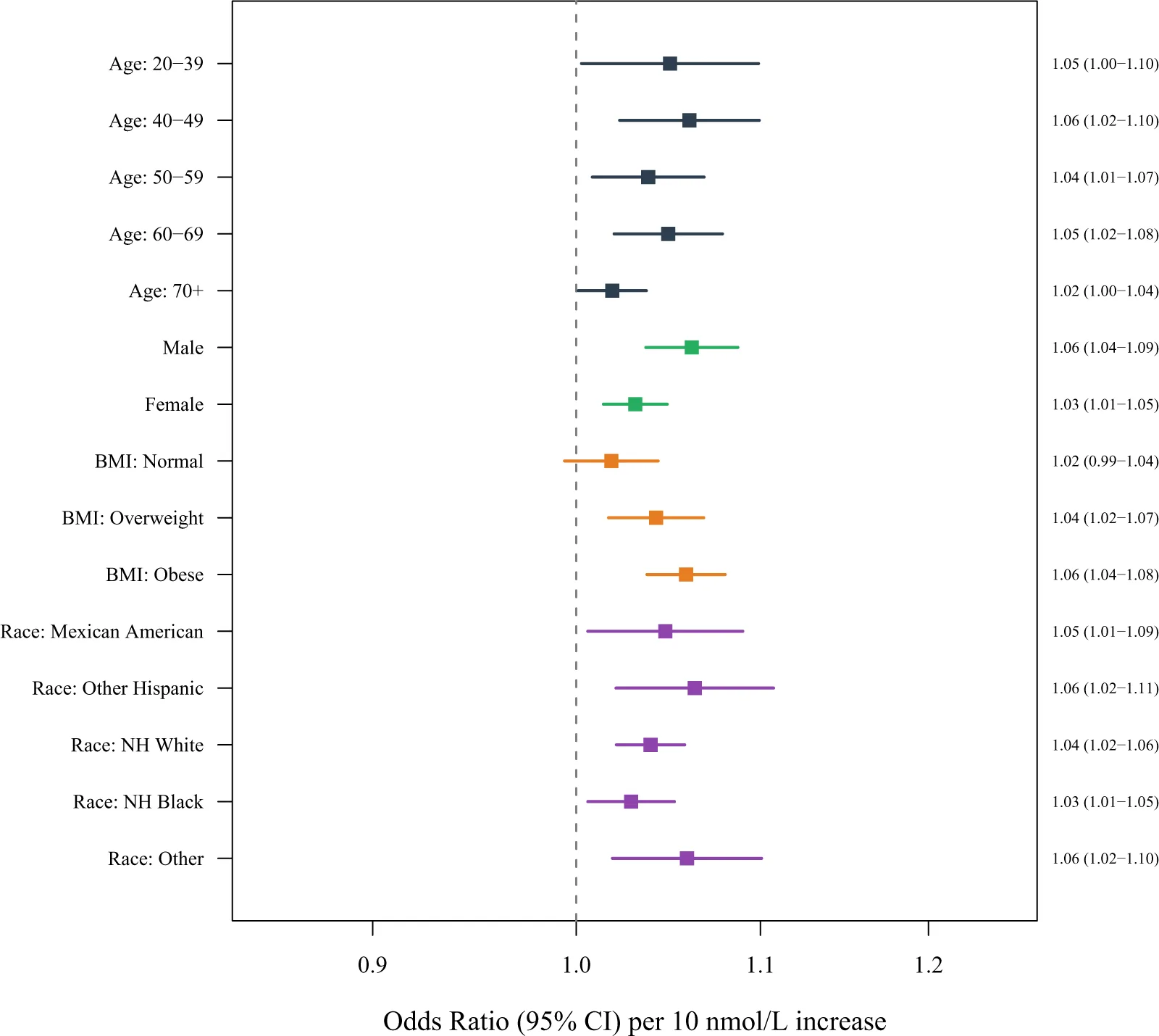
Forest plot of subgroup-specific ORs (per 10 nmol/L increase) for OA, stratified by age, sex, BMI, and race/ethnicity. *P*-values for interaction are shown.

**Table 3:** Subgroup analyses and survey-weighted odds ratios for osteoarthritis per 10 nmol/L increase in serum 25(OH)D across demographic and clinical strata, NHANES 2007–2023 (*N* = 35,887).

Subgroup	OR (95% CI)	*P*-value	*N*
Overall	1.042 (1.027–1.057)	<0.001	35 887
Age: 20-39	1.050 (1.003–1.099)	0.0380	11 459
Age: 40-49	1.060 (1.023–1.099)	0.0017	5842
Age: 50-59	1.038 (1.008–1.068)	0.0121	5807
Age: 60-69	1.049 (1.020–1.079)	0.0011	6557
Age: 70 +	1.019 (1.001–1.037)	0.0383	6222
Male	1.062 (1.037–1.087)	<0.001	17 168
Female	1.031 (1.014–1.048)	<0.001	18 719
BMI: Normal	1.018 (0.994–1.043)	0.1396	10 263
BMI: Overweight	1.042 (1.017–1.068)	0.0012	11 766
BMI: Obese	1.058 (1.037–1.080)	<0.001	13 858
Race: Mexican American	1.047 (1.006–1.090)	0.0248	4973
Race: Other Hispanic	1.063 (1.021–1.108)	0.0036	3785
Race: NH White	1.039 (1.021–1.058)	<0.001	15 788
Race: NH Black	1.029 (1.006–1.052)	0.0134	6911
Race: Other	1.059 (1.019–1.101)	0.0040	4430

#### Age

The association was observed across all age groups. The strongest association was observed among adults aged 40–49 years (OR = 1.060; 95% CI: 1.023–1.099; *P* = 0.002), whereas the weakest was among those aged ≥70 years (OR = 1.019; 95% CI: 1.001–1.037; *P* = 0.038). A statistically significant interaction between vitamin D and age was detected (*P* for interaction < 0.001), suggesting that the magnitude of the association decreases with advancing age.

#### Sex

The association was significant in both males (OR = 1.062; 95% CI: 1.037–1.087; *P* < 0.001) and females (OR = 1.031; 95% CI: 1.014–1.048; *P* < 0.001). The vitamin D–sex interaction was not statistically significant (*P* for interaction = 0.079), suggesting no meaningful effect modification by sex.

#### BMI

A noteworthy gradient was observed across BMI categories. The association was strongest among obese participants (BMI ≥30 kg/m^2^; OR = 1.058; 95% CI: 1.037–1.080; *P* < 0.001) and moderately strong among overweight participants (BMI 25–30; OR = 1.042; 95% CI: 1.017–1.068; *P* = 0.001), but was not statistically significant among normal-weight participants (BMI <25; OR = 1.018; 95% CI: 0.994–1.043; *P* = 0.140). The vitamin D–BMI interaction was statistically significant (*P* for interactio*n* = 0.004), indicating that excess adiposity amplifies the vitamin D–OA association.

#### Race/ethnicity

The association was consistently positive across all five racial/ethnic groups. The strongest associations were observed among Other Hispanic (OR = 1.063; 95% CI: 1.021–1.108; *P* = 0.004) and Other/Multi-racial participants (OR = 1.059; 95% CI: 1.019–1.101; *P* = 0.004). Non-Hispanic White participants exhibited an OR of 1.039 (95% CI: 1.021–1.058; *P* < 0.001), Non-Hispanic Black participants had an OR of 1.029 (95% CI: 1.006–1.052; *P* = 0.013), and Mexican American participants had an OR of 1.047 (95% CI: 1.006–1.090; *P* = 0.025).

### Sensitivity analyses

The primary findings were robust across all sensitivity analyses (Table 4). Restricting the sample to adults aged ≥50 years yielded a comparable OR of 1.036 (95% CI: 1.019–1.053; *P* < 0.001; *n* = 18 586). Among young adults aged 20–39 years, the association remained significant (OR = 1.059; 95% CI: 1.012–1.109; *P* = 0.014; *n* = 11 459). Excluding extreme vitamin D values (>125 nmol/L) did not materially alter the results (OR = 1.055; 95% CI: 1.036–1.075; *P* < 0.001; *n* = 34 305). Restricting to the physiological range of 25–100 nmol/L strengthened the association slightly (OR = 1.072; 95% CI: 1.047–1.098; *P* < 0.001; *n* = 29 974). The association was consistent when restricted to adults aged ≥40 years (OR = 1.040; 95% CI: 1.025–1.056; *P* < 0.001; *n* = 24 428), among non-Hispanic White participants only (OR = 1.039; 95% CI: 1.021–1.058; *P* < 0.001; *n* = 15 788), and after excluding the most recent 2021–2023 cycle (OR = 1.041; 95% CI: 1.022–1.060; *P* < 0.001; *n* = 30 376).

**Table 4:** Sensitivity analyses assessing the association between serum 25(OH)D (per 10 nmol/L increase) and osteoarthritis prevalence under varying inclusion criteria and model specifications, NHANES 2007–2023.

Analysis	OR (95% CI)	*P*-value	*N*
Restricted to adults ≥50	1.036 (1.019–1.053)	<0.001	18 586
Age: 20–39 years	1.059 (1.012–1.109)	0.0142	11 459
Exclude Vitamin D >125 nmol/L	1.055 (1.036–1.075)	<0.001	34 305
Vitamin D 25–-100 nmol/L only	1.072 (1.047–1.098)	<0.001	29 974
Adults ≥40 (comparable)	1.040 (1.025–1.056)	<0.001	24 428
NH White only	1.039 (1.021–1.058)	<0.001	15 788
Excluding 2021–2023	1.041 (1.022–1.060)	<0.001	30 376

### Assessment of Unmeasured Confounding

E-value analysis was conducted to quantify the potential impact of unmeasured confounding. For the fully adjusted estimate (OR = 1.053; 95% CI: 1.037–1.069), the E-value for the point estimate was 1.29, and for the lower bound of the confidence interval, it was 1.23. This indicates that an unmeasured confounder would need to be associated with both serum 25(OH)D and OA by a risk ratio of at least 1.29, above and beyond the measured covariates, to explain away the observed association. While the E-value is modest, reflecting the moderate effect size, the consistency of the association across multiple subgroups and sensitivity analyses supports the robustness of the findings.

### Inflammatory mediation analysis

We evaluated whether C-reactive protein (CRP), a systemic marker of inflammation, mediated the association between vitamin D and OA using the Baron–Kenny causal steps approach among 15 398 participants with available CRP data. The total effect of vitamin D on OA (Step 1) was OR = 1.051. The association between vitamin D and log-transformed CRP (Step 2) was non-significant (β = −0.0084; *P* = 0.093). After adjusting for CRP, the direct effect of vitamin D on OA (Step 3) was OR = 1.054, and the association between CRP and OA was significant (OR = 1.177). The estimated percentage mediated by CRP was −4.7%, indicating that CRP does not act as a meaningful intermediary in the vitamin D–OA pathway. The negative mediation percentage suggests possible inconsistent mediation or suppression effects. Collectively, these results indicate that the vitamin D–OA association is not primarily driven by systemic inflammation as captured by CRP.

### Temporal trends across NHANES cycles

The association between serum 25(OH)D and OA was examined separately within each of the seven NHANES cycles to assess temporal consistency (Fig. 3). The cycle-specific ORs ranged from 1.032 (2009–2010; 95% CI: 1.005–1.060) to 1.088 (2017–2018; 95% CI: 1.036–1.142). The association was statistically significant in five of the seven cycles, with two cycles (2011–2012: OR = 1.035, 95% CI: 0.974–1.098; and 2015–2016: OR = 1.038, 95% CI: 0.991–1.086) that showed borderline significance, likely due to smaller within-cycle sample sizes.

**Figure 3.**
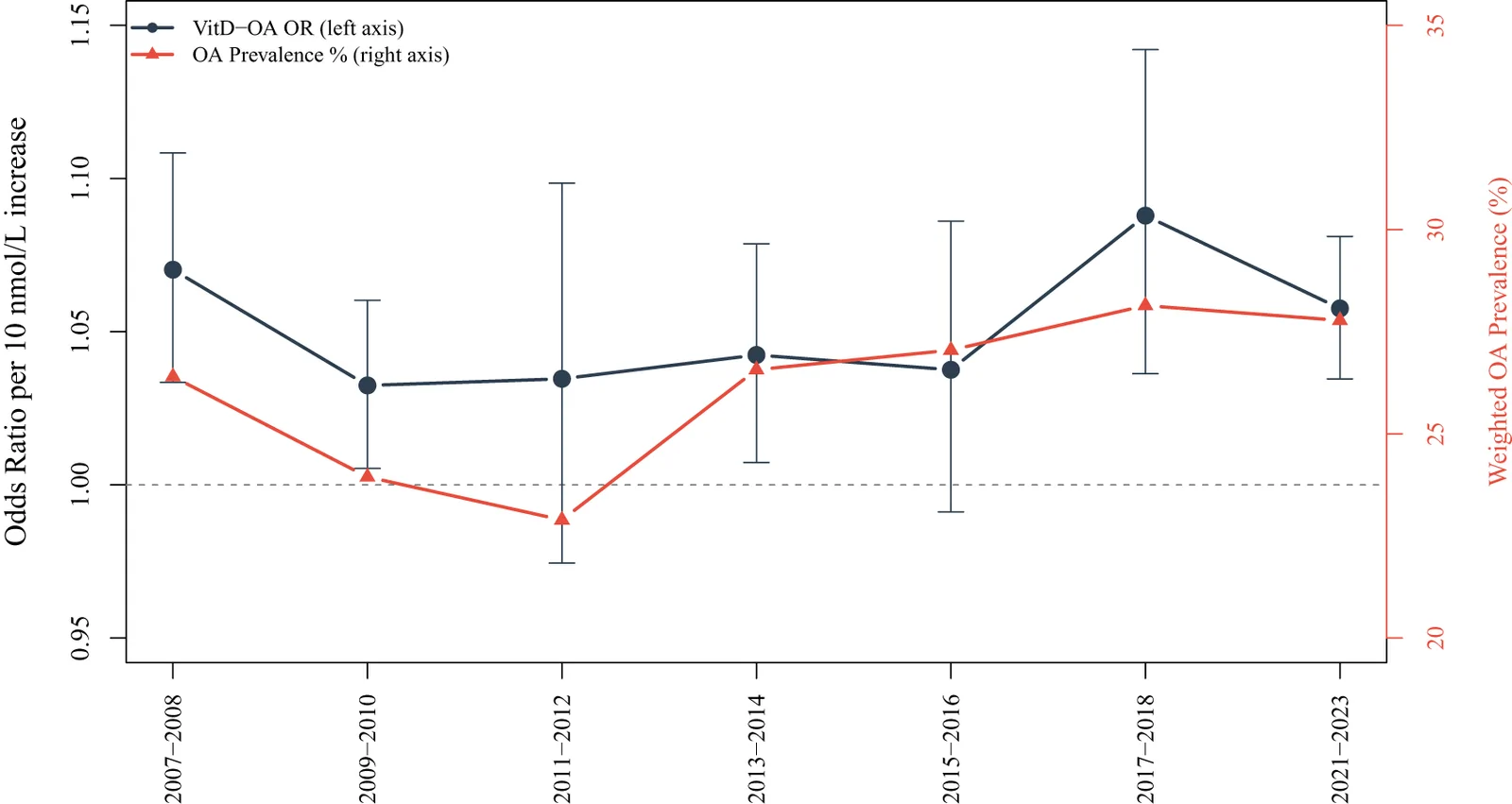
Temporal trends across seven NHANES cycles (2007–2023). Each point represents a cycle-specific OR with a 95% CI.

Over the study period, the mean serum 25(OH)D concentration increased from 67.3 nmol/L in 2007–2008 to 79.0 nmol/L in 2021–2023, paralleling national trends in vitamin D supplementation. The weighted OA prevalence fluctuated modestly, ranging from 22.9% (2011–2012) to 28.1% (2017–2018). Importantly, the positive direction of the vitamin D–OA association was maintained across all cycles spanning a 16-year period, supporting the temporal stability and external validity of the primary findings.

### Summary of key findings

In summary, this nationally representative cross-sectional study of 35 887 U.S. adults demonstrated a significant, positive, and non-linear association between serum 25(OH)D concentrations and the prevalence of OA. Each 10 nmol/L increase in 25(OH)D was associated with 4.2–5.3% higher odds of OA after multivariable adjustment. The association exhibited a threshold effect, becoming clinically meaningful above 50 nmol/L and plateauing near 125 nmol/L. The relationship was consistent across age, sex, race, and BMI subgroups, though it was significantly modified by age (*P* < 0.001) and BMI (*P* = 0.004). CRP did not mediate the association, and the findings were robust across seven sensitivity analyses and seven NHANES cycles spanning 2007–2023.

## Discussion

### Summary of principal findings

In this nationally representative cross-sectional study of 35,887 US adults across seven NHANES cycles (2007–2023), we found a significant positive association between serum 25(OH)D concentration and the prevalence of self-reported OA. In the fully adjusted model, a 10 nmol/L increase in serum 25(OH)D concentration was associated with 5.3% higher odds of OA (OR = 1.053; 95% CI: 1.037–1.069). The dose–response relationship was nonlinear according to piecewise-linear calculations, with a threshold below 50 nmol/L and an upper plateau at approximately 100–125 nmol/L. The association was consistent across subgroups, robust to seven sensitivity analyses as well as all survey cycles, and the effect was not mediated by C-reactive protein (CRP) levels.

### Comparison with existing literature

Our results align with the cross-sectional analysis by Yu *et al*, who found a positive correlation between high serum 25(OH)D and OA prevalence based on NHANES data (2001–2018), where the odds ratios for serum 25(OH)D levels of 50–75, 75–100, and ≥100 nmol/L were reported to be associated with increased OA prevalence [OR: 1.25 (95% CI: 0.91–1.73), OR: 1.62 (95% CI: 0.90–2.80), OR: 1.91 (95% CI: N/A)] compared with <50 nmol/L^[^1^]^. Our study builds on these results in several critical ways: we included younger adults (≥20 years), used only the standardized LC-MS/MS assay across all cycles, explored non-linear dose–response via RCSs, performed mediation analyses using an inflammation index, conducted formal tests of mediation, and examined temporal consistency over seven survey cycles.

The positive association observed between vitamin D status and OA pathogenesis may conflict with the current understanding in the field, which suggests a role for vitamin D insufficiency as a contributor to OA pathogenesis. Several longitudinal studies have reported an association between low serum 25(OH)D levels and the risk of radiographic knee OA progression and cartilage volume loss^[^7,10^]^. Cao *et al* conducted a systematic review and found moderate evidence associating low 25(OH)D levels with cartilage loss when measuring joint space narrowing and cartilage volume^[^7^]^. Additionally, according to a meta-analysis, vitamin D deficiency was reported in 56.7% of patients with knee OA^[^12^]^. Our analyses were cross-sectional, whereas the other studies were longitudinal and focused mainly on structural progression rather than on the prevalence of OA in terms of its occurrence (which delineates a different epidemiological question).

Our findings are noteworthy because they align with the consistent null results observed in randomized controlled trials (RCTs) of vitamin D supplementation for OA treatment. McAlindon *et al* showed in a 2-year double-blind RCT that plain vitamin D supplementation (2000 IU/d) did not reduce knee pain or loss of cartilage volume versus placebo^[^20^]^. Similarly, Jin *et al* found no overall effect of vitamin D supplementation on tibial cartilage volume^[^13^]^. Arden *et al* also found no benefit of vitamin D supplementation on knee OA progression in the three-year VIDEO trial^[^21^]^. These findings regarding the lack of evidence for benefit in RCTs, in addition to our cross-sectional observation, point toward a relationship between vitamin D and OA that is unlikely to be simply protective and potentially reflects reverse causation or confounding due to the health-seeking behavior of patients with OA.

### Potential mechanisms

The paradoxical positive relationship between increased 25(OH)D levels and OA prevalence may be explained by several mechanisms, including the following: First, reverse causation is a possible explanation: patients diagnosed with OA may be more likely to pursue vitamin D supplementation as part of self-initiated or physician-recommended management strategies and thus have higher serum 25(OH)D levels^[^22^]^. The NHANES database does not have granular data on vitamin D supplementation that would allow complete adjustment for this behavior, and the growing public awareness of vitamin D benefits has driven considerable expansion in supplement use during the study period. In fact, the use of vitamin D supplements among U.S. adults sharply increased from 5.1% in 1999–2000 to 19.0% in 2011–2012^[^23^]^.

This interpretation is consistent with the temporal trend in our data, which revealed increases in the mean 25(OH)D level (from 61.5 to 81.8 nmol/L) and in OA prevalence (from 26.9% to 33.6%) across survey cycles.

Second, the observed association may involve biological mechanisms. Vitamin D regulates osteoblast and osteoclast activity; thus, it can modulate subchondral bone remodeling^[^6^]^.

Vitamin D has anti-inflammatory and chondroprotective effects in various cell types, including the inhibition of MMPs (MMP-1, -3, -9, and -13) and the inhibition of signaling pathways activated by pro-inflammatory cytokines in chondrocytes^[^6^]^, which is likely counterbalanced by increased subchondral bone turnover as circulating concentrations rise. According to Garfinkel *et al*, 1α,25(OH)_2_D_3_ signaling is a potential chondroprotective pathway; however, it can also activate signaling cascades associated with chondrocyte hypertrophy implicated in OA pathogenesis^[^6^]^. This dual action of vitamin D on joint tissues may explain why both vitamin D deficiency and excess could theoretically have deleterious effects on joint health.

Third, the association may be partially due to confounding factors such as healthcare access and behavior. Higher socioeconomic status and frequent encounters with the health system also lead to higher vitamin D levels (through vitamin D supplementation or dietary intake) and clinical diagnoses of OA^[^22^]^. Although we controlled for the poverty–income ratio, residual confounding by patterns of healthcare utilization cannot be completely excluded.

### Subgroup analyses and effect modification

There was a significant interaction with BMI (*P* = 0.004). The association between 25(OH)D and OA was strongest in obese individuals (OR = 1.058) and was statistically insignificant in individuals with normal weight (OR = 1.018; *P* = 0.140). This finding is consistent with that of Yu *et al*, who also found that BMI was a significant modifier^[^14^]^. Obesity is recognized as a metabolic risk factor for OA in addition to its effects on mechanical loading^[^24^]^.

Adipose tissue acts as an endocrine organ and releases pro-inflammatory adipokines, such as leptin, which upregulate MMPs^[^24^]^ and pro-inflammatory cytokines (IL-1β, IL-6, and TNF-α) in chondrocytes^[^25^]^. Higher vitamin D levels in the presence of obesity-related metabolic dysfunction may create a dilemma in which vitamin D's net positive effect on joint tissues transitions toward an adverse effect through potentiation of subchondral bone remodeling in already inflamed environments.

Differences in vitamin D supplementation patterns relative to OA symptom onset could explain the differences observed between age groups, as indicated by the large age interaction (*P* < 0.001), with the strongest association among those aged 40–49 years (OR = 1.060). This age group also corresponds to the peak onset of symptomatic OA^[^26^]^, and seeking care for early-stage joint symptoms may be positively related to increased supplement use in this age group. The weaker association in older adults (≥70 years; OR = 1.019) may indicate survivorship effects or that age-related cartilage degeneration has become the predominant factor^[^26^]^, undermining the vitamin D–OA relationship. The absence of a significant sex interaction (*P* = 0.079) indicates that the association proceeds through pathways common to both sexes.

### Inflammatory mediation analysis

Our formal mediation analysis showed that CRP did not meaningfully mediate the 25(OH)D–OA association (estimated mediation: -4.7%), and that the vitamin D–to–CRP path was not significant (β = −0.0084; *P* = 0.093). This is an important finding because it suggests that the positive association of Mz-EMR2 was not mediated by systemic inflammation. Increased CRP levels have been associated with OM – which has been reported to be reduced in patients with OA compared with normal subjects^[^27^]^ – and vitamin D plays an immunomodulatory role by activating anti-hyperimmune functions^[^28^]^. Nevertheless, OA is increasingly acknowledged as a predominantly local inflammatory disease process, as opposed to systemic^[^28^]^. The lack of CRP-associated effects indicates that the 25(OH)D–OA association may operate through non-inflammatory pathways, including those linked to bone remodeling, behavioral factors (i.e., supplement use), or residual confounding factors, such as physical activity.

### Assessment of unmeasured confounding

The E-value for the fully adjusted OR of 1.053 was 1.29 (point estimate) and 1.2 (lower bound of the confidence interval). This means that for an unmeasured confounder to completely account for the observed association, it would need to be associated with both serum 25(OH)D and OA, with a risk ratio of at least 1.29^[^18^]^. Although this is moderately robust, plausible unmeasured confounders (e.g., vitamin D supplementation, patterns of physical activity, frequency of healthcare utilization, and sun exposure) suggest that the combined effect of these unmeasured behavioral and clinical confounders could plausibly explain away the observed association. The E-value analysis highlights the importance of carefully considering causal inferences based on our findings.

### Temporal trends

The consistency of the association across all seven NHANES cycles (cycle-specific ORs 1.032–1.088) reinforces the robustness of our findings and argues against a period-effect artifact. The simultaneous rise in mean serum 25(OH)D (from 61.5 to 81.8 nmol/L) and OA prevalence (from 26.9% to 33.6%) by cycle follows the increased use of vitamin D supplements among the US population^[^23^]^. This temporal pattern is consistent with either a true biological association or, more likely, population-level confounding due to increased dietary supplement use among individuals with OA or those at risk of developing it. To the best of our knowledge, this is the first study to systematically assess the temporal consistency of the 25(OH)D–OA association across multiple NHANES cycles.

### Strengths

This study had some limitations. First, we characterized a large, nationally representative sample (*n* = 35 887), which improved the generalizability of our findings to the noninstitutionalized U.S. adult population. Second, all participating NHANES cycles (2007–2023) used the standardized LC-MS/MS method for 25(OH)D measurement, which was calibrated to the National Institute of Standards and Technology (NIST) and Vitamin D Standardization Program (VDSP)-derived standards, which provide cross-cycle comparability and reduce heterogeneity owing to assay differences. Third, we used several complementary modeling approaches (continuous, quartile, and RCS models) to comprehensively characterize the exposure–outcome association. Fourth, we performed seven sensitivity analyses, as well as formal mediation analysis, E-value assessment, and temporal trend evaluation to provide a comprehensive appraisal of robustness. Relatedly, we included middle-aged adults (aged ≥20 years), thus extending the age range beyond that of previous studies, which were limited to older adults^[^1,14^]^.

### Limitations

This study had several limitations. First, the cross-sectional design does not allow for causal inference; thus, it is impossible to ascertain the temporal relationship between vitamin D levels and OA onset. Reverse causation, whereby individuals with OA increase supplement use, remains a potential alternative explanation that cannot be excluded^[^22^]^. Second, the diagnosis of OA was based on self-report of a physician’s diagnosis of general arthritis (“Has a doctor or other health professional ever told you that you had arthritis?”). While OA represents the overwhelming majority of arthritis cases among adults, particularly in older and obese populations, the lack of radiographic confirmation or specific subtyping introduces a risk of outcome misclassification. The inclusion of rheumatoid arthritis or other inflammatory arthritides in the “arthritis” cohort cannot be entirely excluded, and this limitation should be considered when interpreting the results. Third, critical potential confounders such as granular data on vitamin D supplementation dosage and duration, physical activity levels, sun exposure, dietary intake, co-occurring osteoporosis, and detailed healthcare utilization metrics were not adjusted for in our models due to data unavailability across all pooled cycles. The absence of these variables, particularly supplement use, represents a major limitation, as they could independently drive the observed associations. Fourth, serum 25(OH)D levels were measured at a single time point and may not necessarily reflect long-term vitamin D status. Fifth, CRP data were only available for a subgroup of participants (*n* = 15 398), possibly limiting the power and generalizability of our mediation analysis. Sixth, although we accounted for major confounders such as smoking, diabetes, and poverty–income ratio at baseline, unmeasured confounding cannot be excluded, as reflected by the modest E-values.

### Clinical implications and future directions

Our findings have several important practical implications for managers. Clinically, the positive association between higher serum 25(OH)D levels and OA prevalence suggests that caution should be exercised in assuming that vitamin D supplementation universally supports joint health in patients with OA. This explanation is consistent with the null results of several vitamin D supplementation trials in OA^[^13,20,21^]^. From a research perspective, Mendelian randomization (MR) studies can provide insights into the causal relationships between these factors. Notably, Bergink *et al* applied a two-sample MR design and detected no causal relationship between genetically determined 25(OH)D concentrations and knee OA (OR, 1.03; 95% CI: 0.84–1.26) or hip OA (OR, 1.06; 95% CI: 0.83–1.35)^[^29^]^. This null MR finding supports our interpretation that the positive cross-sectional association is likely due to reverse causation or confounding rather than a true causal effect of vitamin D on OA risk. Large-scale prospective cohort studies with repeated 25(OH)D assessments, detailed supplement information, and radiographic manifestations of OA are required to disentangle the temporal and causal nature of this association. This finding in a dose–response analysis should be highlighted, as it shows a threshold effect above 50 nmol/L; however, further longitudinal studies are required before defining clinical target ranges for musculoskeletal health. Furthermore, it is important to emphasize that while the observed association is highly statistically significant due to the large population sample size, the effect size is small (OR = 1.053 per 10 nmol/L increase). Therefore, the clinical relevance of this association at the individual patient level remains modest, further underscoring the need to interpret these findings without overestimating the clinical impact.

## Conclusion

In this nationally representative study of 35 887 US adults from seven cycles of NHANES (2007–2023), higher serum 25(OH)D concentrations were significantly and positively associated with the prevalence of self-reported OA in the US. The association was non-linear, with a threshold above 50 nmol/L, and persisted across demographic subgroups, sensitivity analyses, and survey cycles, and was not mediated by systemic inflammation (CRP). These results contradict the usual view that higher vitamin D levels protect against OA, strongly pointing toward the role of reverse causation and health-seeking behaviors among diagnosed patients. Further prospective and Mendelian randomization studies are needed to clarify the nature of this association and inform clinical guidelines regarding vitamin D supplementation for musculoskeletal health in older adults.

## Data Availability

NHANES data are publicly available at https://www.cdc.gov/nchs/nhanes/.

## Research registration unique identifying number (UIN)

Not applicable (secondary analysis of publicly available data).

## References

[1] GBD 2021 Osteoarthritis Collaborators. Global, regional, and national burden of osteoarthritis, 1990-2020. Lancet Rheumatol 2023;5:e508–e522. 10.1016/S2665-9913(23)00163-7 PMC10477960 37675071

[2] Hunter DJ Bierma-zeinstra S. Osteoarthritis. Lancet 2019;393:1745–59. 10.1016/S0140-6736(19)30417-9 31034380

[3] Martel-pelletier J Barr AJ Cicuttini FM. Osteoarthritis. Nat Rev Dis Primers. 2016;2:16072. 10.1038/nrdp.2016.72 27734845

[4] Holick MF. Vitamin D deficiency. N Engl J Med 2007;357:266–81. 10.1056/NEJMra070553 17634462

[5] Institute of Medicine. Dietary Reference Intakes for Calcium and Vitamin D. Washington, DC: The National Academies Press; 2011. 21796828

[6] Garfinkel RJ Dilisio MF Agrawal DK. Vitamin D and its effects on articular cartilage and osteoarthritis. Orthop J Sports Med 2017;5:2325967117711376. 10.1177/2325967117711376 PMC5480771 28680892

[7] Cao Y Winzenberg T Nguo K. Association between serum levels of 25-hydroxyvitamin D and osteoarthritis. Rheumatology (Oxford). 2013;52:1323–34. 10.1093/rheumatology/ket132 23542678

[8] Forrest KY Stuhldreher WL. Prevalence and correlates of vitamin D deficiency in US adults. Nutr Res 2011;31:48–54. 10.1016/j.nutres.2010.12.001 21310306

[9] Schleicher RL Sternberg MR Lacher DA. The vitamin D status of the US population from 1988 to 2010. Am J Clin Nutr. 2016;104:454–61. 10.3945/ajcn.115.127985 PMC4962157 27385610

[10] Zhang FF Driban JB Lo GH. Vitamin D deficiency is associated with progression of knee osteoarthritis. J Nutr. 2014;144:2002–08. 10.3945/jn.114.193227 PMC4230211 25411034

[11] Ding C Cicuttini F Parameswaran V. Serum levels of vitamin D, falling and progression of knee osteoarthritis: a two-year longitudinal study. QJM 2009;102:521–8.

[12] Iqhrammullah M Wira JF Nababan SP. Global prevalence of vitamin D deficiency among patients with knee osteoarthritis: a systematic review and meta-analysis. Nutr Health 2026. doi:10.1177/02601060251399818. 40842280

[13] Jin X Jones G Cicuttini F. Effect of vitamin D supplementation on tibial cartilage volume and knee pain. JAMA. 2016;315:1005–13. 10.1001/jama.2016.1961 26954409

[14] Yu G Lin Y Dai H. Association between serum 25-hydroxyvitamin D and osteoarthritis: NHANES 2001-2018. Front Nutr. 2023;10:1016809. 10.3389/fnut.2023.1016809 PMC10011108 36925955

[15] Centers for Disease Control and Prevention. National Health and Nutrition Examination Survey (NHANES). Accessed 15 February 2026. https://www.cdc.gov/nchs/nhanes/index.htm

[16] Lumley T. Analysis of complex survey samples. J Stat Softw 2004;9:1–19.

[17] Harrell FE Jr. Regression Modeling Strategies. 2nd ed. Cham: Springer; 2015.

[18] VanderWeele TJ Ding P. Sensitivity analysis in observational research: introducing the E-value. Ann Intern Med 2017;167:268–74. 10.7326/M16-2607 28693043

[19] Baron RM Kenny DA. The moderator-mediator variable distinction. J Pers Soc Psychol 1986;51:1173–82. 10.1037//0022-3514.51.6.1173 3806354

[20] McAlindon T LaValley M Schneider E. Effect of vitamin D supplementation on knee pain and cartilage volume loss. JAMA. 2013;309:155–62. 10.1001/jama.2012.164487 PMC3984919 23299607

[21] Arden NK Cro S Sheard S. Vitamin D supplementation on knee osteoarthritis, the VIDEO study. Osteoarthritis Cartilage. 2016;24:1858–66. 10.1016/j.joca.2016.05.020 PMC5045720 27264058

[22] Autier P Boniol M Pizot C. Vitamin D status and ill health. Lancet Diabetes Endocrinol 2014;2:76–89. 10.1016/S2213-8587(13)70165-7 24622671

[23] Kantor ED Rehm CD Du M. Trends in dietary supplement use among US adults. JAMA. 2016;316:1464–74. 10.1001/jama.2016.14403 PMC5540241 27727382

[24] Francisco V Perez T Pino J. Biomechanics, obesity, and osteoarthritis. J Orthop Res. 2018;36:594–604. 10.1002/jor.23788 29080354

[25] Pearle AD Scanzello CR George S. Elevated CRP levels in OA. Osteoarthritis Cartilage. 2007;15:516–23. 10.1016/j.joca.2006.10.010 17157039

[26] Loeser RF Collins JA Diekman BO. Ageing and the pathogenesis of osteoarthritis. Nat Rev Rheumatol 2016;12:412–20. 10.1038/nrrheum.2016.65 PMC4938009 27192932

[27] Karvonen-Gutierrez CA Harlow SD Jacobson J. Longitudinal serum leptin and knee joint damage. Ann Rheum Dis. 2014;73:883–89. 10.1136/annrheumdis-2012-202685 PMC3884071 23576710

[28] Robinson WH Lepus CM Wang Q. Low-grade inflammation in OA pathogenesis. Nat Rev Rheumatol. 2016;12:580–92. 10.1038/nrrheum.2016.136 PMC5500215 27539668

[29] Bergink AP Trajanoska K Uitterlinden AG. Mendelian randomization study on vitamin D levels and osteoarthritis risk: a concise report. Rheumatology (Oxford). 2021;60:4982–84. 10.1093/rheumatology/keaa697 33463692

